# Cuproptosis-related signature predicts prognosis, immunotherapy efficacy, and chemotherapy sensitivity in lung adenocarcinoma

**DOI:** 10.3389/fonc.2023.1127768

**Published:** 2023-03-15

**Authors:** Gujie Wu, Qin Hu, Hongyu Chen, Min He, Huiyun Ma, Lin Zhou, Kun Xu, Hefei Ren, Juntao Qi

**Affiliations:** ^1^ Research Center of Clinical Medicine, Affiliated Hospital of Nantong University, Nantong, Jiangsu, China; ^2^ Research Center of Clinical Medicine, Shenzhen Hospital of Shanghai University of Traditional Chinese Medicine, Shenzhen, China; ^3^ Department of Laboratory Medicine, Changzheng Hospital, Naval Medical University, Shanghai, China

**Keywords:** cuproptosis, lung adenocarcinoma, prognosis, tumor microenvironment, immunotherapy

## Abstract

**Background:**

Cuproptosis is a novel form of programmed cell death that disrupts the tricarboxylic acid (TCA) cycle and mitochondrial function. The mechanism of cuproptosis is quite different from that of common forms of cell death such as apoptosis, pyroptosis, necroptosis, and ferroptosis. However, the potential connection between cuproptosis and tumor immunity, especially in lung adenocarcinoma (LUAD), is poorly understood.

**Methods:**

We used machine learning algorithms to develop a cuproptosis-related scoring system. The immunological features of the scoring system were investigated by exploring its association with clinical outcomes, immune checkpoint expression, and prospective immunotherapy response in LUAD patients. The system predicted the sensitivity to chemotherapeutic agents. Unsupervised consensus clustering was performed to precisely identify the different cuproptosis-based molecular subtypes and to explore the underlying tumor immunity.

**Results:**

We determined the aberrant expression and prognostic relevance of cuproptosis-related genes (CRGs) in LUAD. There were significant differences in survival, biological function, and immune infiltration among the cuproptosis subtypes. In addition, the constructed cuproptosis scoring system could predict clinical outcomes, tumor microenvironment, and efficacy of targeted drugs and immunotherapy in patients with LUAD. After validating with large-scale data, we propose that combining the cuproptosis score and immune checkpoint blockade (ICB) therapy can significantly enhance the efficacy of immunotherapy and guide targeted drug application in patients with LUAD.

**Conclusion:**

The Cuproptosis score is a promising biomarker with high accuracy and specificity for determining LUAD prognosis, molecular subtypes, immune cell infiltration, and treatment options for immunotherapy and targeted therapies for patients with LUAD. It provides novel insights to guide personalized treatment strategies for patients with LUAD.

## Introduction

Lung cancer is the most common malignancy and the leading cause of cancer-related deaths worldwide (1). Alarmingly, the incidence and mortality of lung cancer continue to grow (2). Of all non-small cell lung cancers, lung adenocarcinoma (LUAD) has received much attention as it is the most common histological subtype (3). Despite recent advances in chemotherapy, radiotherapy, and targeted therapy, only 15% of patients with LUAD survive over 5 years (4, 5). Therefore, better prognostic tools and biomarkers accurately predicting the characteristics of tumors are urgently needed to stratify patients and personalize treatment strategies for LUAD.

Cuproptosis is a recently discovered form of cell death that differs from oxidative stress-related cell death (such as ferroptosis, apoptosis, and necroptosis) (6). Cuproptosis is regulated by protein lipoylation; copper binds to lipoylated enzymes of the tricarboxylic acid cycle, leading to subsequent aggregation of lipoylated proteins and loss of iron-sulfur cluster proteins, finally leading to proteotoxic stress and ultimately, cell death (7). As cancer cells are highly proliferative with various dysregulation and heterogeneity, we aimed to investigate whether cuproptosis could provide new opportunities in the research and clinical practice of oncology. Considering that homeostatic dysregulation of copper plays an important role in cancer, cuproptosis induction is a promising new therapeutic approach, especially for tumors that are resistant to conventional treatment modalities (8).

In recent years, researchers have found that targeted therapy combined with immunotherapy has beneficial therapeutic effects and good prospects in patients with advanced LUAD, and have gradually replaced conventional monotherapy with targeted drugs (9). However, there is still a lack of effective molecular phenotypes to identify patients likely to benefit from immunotherapy and to predict the clinical progression of patients with advanced LUAD. Therefore, new molecular phenotypes should be established to precisely identify suitable LUAD populations for personalized therapy.

**Figure 1:**
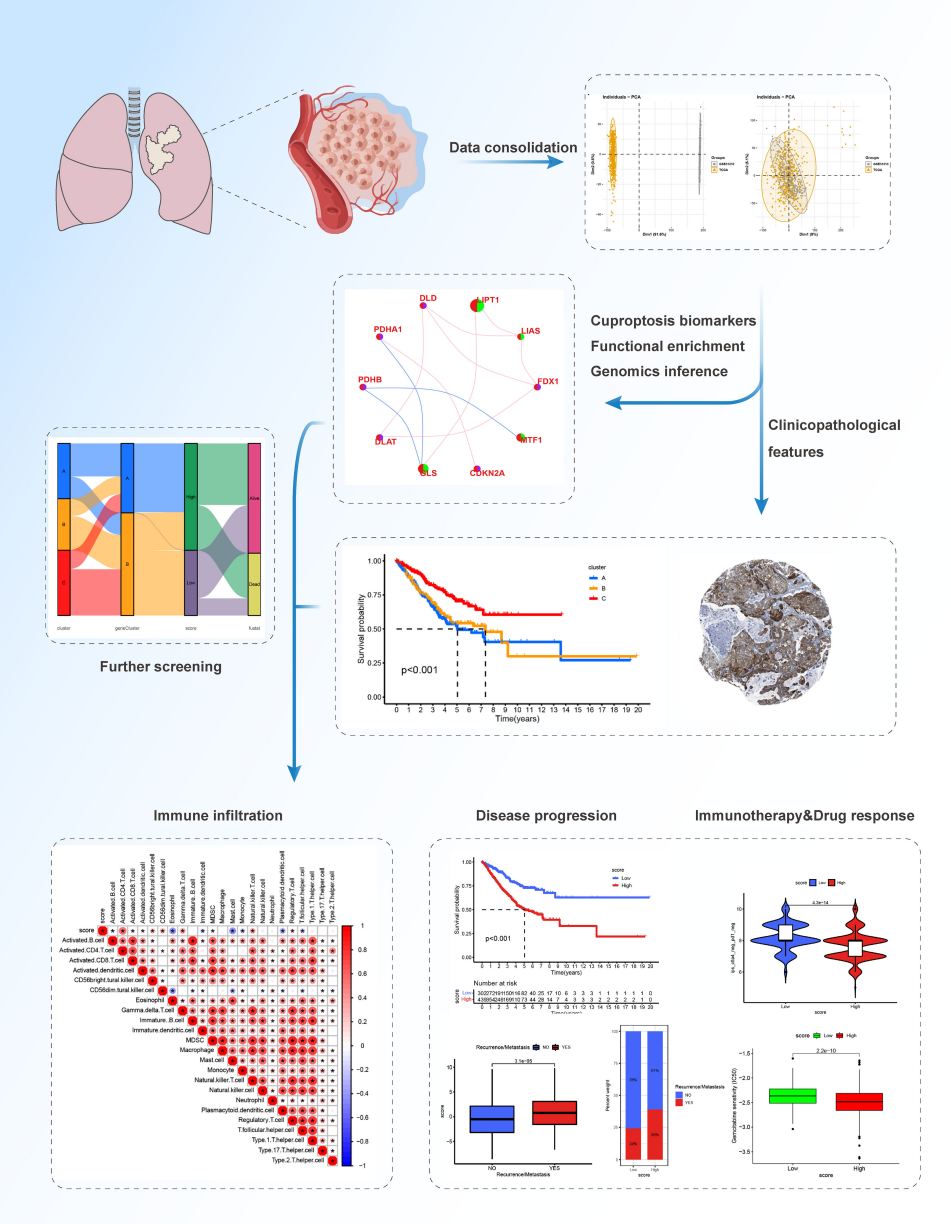
The workflow of the study.

In this study, we comprehensively evaluated the expression profiles of cuproptosis-related genes and explored a comprehensive overview of the intratumoral immune landscape in LUAD. We covered large-scale LUAD cohort of The Cancer Genome Atlas (TCGA) and Gene Expression Omnibus (GEO) databases to establish novel molecular subtypes. We classified LUAD patients with heterogenous cuproptosis status and different clinical outcomes. We further established a novel scoring system, the cuproptosis score, to predict the clinical outcomes, tumor immune microenvironment (TIME), and the efficacy of targeted therapy and immunotherapy in patients with LUAD and explored specific targets and drugs. Our results provide new insights to facilitate personalized therapy for patients with advanced or unresectable LUAD. The workflow of this study is shown in **Figure 1**.

## Materials and methods

### Data source

Data merging was done using the TCGA-LUAD cohort, which included 524 LUAD samples from the TCGA database (https://tcga-data.nci.nih.gov/tcga/), and the GSE31210 cohort, which included 226 LUAD samples from the GEO database (http://www.ncbi.nlm.nih.gov/geo/). Clinical information and normalized matrix files were retrieved from the GEO database, and information on gene expression from RNA sequencing (FPKM values) and clinical information were obtained from TCGA. The FPKM values were then transformed to transcripts per kilobase million (TPM) values for additional analysis, and batch effects were removed using principal component analysis (PCA) and “ComBat” from the “SVA” R package. Samples without complete survival information were disqualified. Finally, we were able to get a complete LUAD cohort with 750 samples and 16928 genes.

### Collection and validation of clinical samples

LUAD tissues were obtained from patients who had undergone surgery at the Affiliated Hospital of Nantong University. In our cohort, 5 pairs of tissues were obtained between 2018 and 2020. The study was authorized by the Ethical Committee of Affiliated Hospital of Nantong University (2022-L119). RNA from tumor tissues was extracted using TRIzol Reagent (Invitrogen), and the cDNA was obtained through reverse transcription using a PrimeScript™ RT Reagent Kit (TaKaRa, Shiga, Japan). Real-time quantitative polymerase chain reaction (qPCR) was conducted in triplicate for each sample using a SYBR Premix Ex Taq II Reagent Kit (TaKaRa). The primer sequences for the target genes were as follows: CDKN2A forward 5′-CCAGGTCATGATGAT-3′, reverse5′-TGCAGCACCACCA-3′; GAPDH forward 5′-TGACTTCAACAGCGACACCCA-3′, reverse 5′-CACCCTGTTGCTGTAGCCAAA-3′.

### Immunohistochemical staining

The Human Protein Atlas (HPA) (http://www.proteinatlas.org/) is a database containing immunohistochemical staining results from a wide range of tumors and normal tissues. We have used HPA data here to explore the expression of gene.

### Gene set cancer analysis database

GSCALite (http://bioinfo.life.hust.edu.cn/web/GSCALite/) provides an online cancer genomic analysis platform by integrating 33 cancers data from TCGA and normal tissue genomics data from GTEx. In this study, we analyzed the genomic level, copy number level, and methylation level of CRGs in LUAD by GSCALite.

### Functional enrichment analysis

Functional enrichment analysis of differentially expressed genes connected to CRGs in LUAD was used to investigate functional annotation and enrichment pathways. ClusterProfiler was used to evaluate Gene Ontology (GO) and Kyoto Encyclopedia of Genes and Genomes (KEGG) pathways.

### Calculation of cuproptosis score

According to the positive and negative relationships between the DEGs and the cluster signature, the DEGs were divided into two groups, namely sigC1 and sigC2. Then, the “clusterProfiler” R package was used for gene annotation. We then used the Boruta algorithm combined with PCA to reduce the dimensionality of the DEGs subgroups and calculated the cuproptosis score for each sample. The LUAD comprehensive cohort was divided into the high- and low-cuproptosis score groups based on the optimal cutoff value. The cuproptosis score of each LUAD sample was calculated using the following formula: Cuproptosis score=∑PsigC1−∑PsigC2

### Clinical subgroup analysis

We selected “survival status”, “T stage”, “N stage”, “M stage”, “clinical stage”, and “recurrence or metastasis” as clinical subgroup characteristics, and drew box plots to show the differences in the cuproptosis score between different clinical characteristics. A stacked histogram was drawn to show the proportion of each clinical characteristic in the high- and low-cuproptosis score groups.

### Prognosis and immune exploration

We used the “survival” and “survminer” R packages to perform survival analysis to compare the differences in OS between the high- and low-cuproptosis score groups, and used the “ggalluvial” R package to draw Sankey diagrams to visualize the correspondence among cuproptosis score groups, different subtypes, and prognosis. Box plots were used to compare the differences in the cuproptosis score of different subtypes. ssGSEA was used to quantify the infiltration abundance of immune cells, and the relationship between cuproptosis score and immune cell infiltration levels was displayed using a correlation heat map.

### Immunotherapy efficacy

We used the “limma” R package to compare the differences in the gene expression of several common immune targets. Next, we downloaded the immunophenoscore (IPS) data of the TCGA-LUAD cohort from The Cancer Immunome Atlas (TCIA) database to explore the differences in the efficacy of the four immune checkpoint inhibitors (ICIs) between the high- and low-cuproptosis groups (10), including ctla4_pos_pd1_pos, ctla4_neg_pd1_pos, ctla4_pos_pd1_neg, and ctla4_neg_pd1_neg. In addition, we collected data from 2 immunotherapy cohorts for immune benefit validation, including the GSE91061 (Nivolumab immunotherapy) and GSE13507 cohorts (intravenous BCG immunotherapy).

### Statistical analysis

All analyses were performed using R version 4.1.1. Unless otherwise specified, Pearson’s correlation coefficient was used for correlation analysis in this study. For comparison between the two groups in the bioinformatics analysis section, the Wilcoxon test was used for difference analysis. For comparison between the two groups in the experimental section, the Students’ t-test was used for difference analysis. Kaplan-Meier survival analysis and log-rank tests were used to compare the survival of the different groups of patients. For all statistical analyses, a two-tailed p <0.05 was considered statistically significant.

## Results

### Genetic and transcriptional alterations of CRGs in LUAD

**Figure 2:**
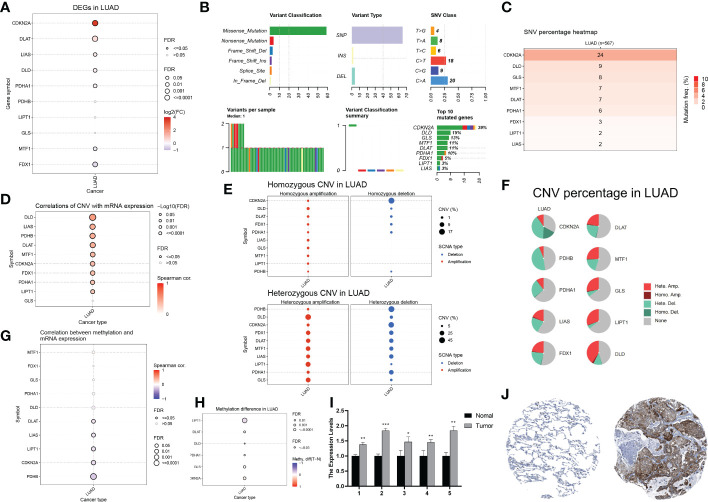
Genetic and transcriptional alterations of CRGs in LUAD **(A)** Differential gene expression of CRGs in LUAD (Solid circles indicate differential expression, red is high tumour expression). **(B)** Variant classification, variant type, SNV category, variants per sample, summary of variant classification, and information of CRGs in LUAD. **(C)** Frequency of deleterious mutations in LUAD. **(D)** The correlation between CNV with CRGs expression in LUAD. **(E)** The profile of homozygous CNV and heterozygous CNV of CRGs in LUAD. **(F)** A Pie plot summarizes the CNV of CRGs in LUAD. **(G)** The correlation between methylation with CRGs expression in LUAD. **(H)** Summary of the methylation difference between tumour and normal samples of CRGs in LUAD. **(I)** The mRNA levels of CDKN2A in 5 pairs of clinical samples were confirmed by RT-qPCR. **(J)** Immunohistochemical staining results of CDKN2A in normal and tumor tissues.

First, we analyzed the transcriptomic data of patients with LUAD from TCGA database. The expression of seven cuproptosis-associated genes (CRGs) was found to be significantly higher in LUAD than in normal controls. These seven CRGs, namely, *CDKN2A*, *DLAT*, *LIAS*, *DLD*, *PDHA1*, *MTF1*, and *FDX1*, were deemed differentially expressed genes (DEGs) (**Figure 2A**). Of the seven genes, five (*CDKN2A*, *DLAT*, *LIAS*, *DLD*, and *PDHA1*) were upregulated, while two (*MTF1* and *FDX1*) were downregulated. Gene mutations and copy number variants (CNV) are closely associated with tumorigenesis and tumor progression. Therefore, we screened LUAD patients for genetic changes in the CRGs. **Figure 2B** shows the variant classification, variant type, single nucleotide variant (SNV) class, and variation per sample for the CRGs. We observed that missense mutations were the most common type of genomic mutations and that *CDKN2A*, with a mutation frequency of 39%, harbored the most mutations of all CRGs in LUAD samples. Furthermore, *CDNK2A* also harbored the highest deleterious SNVs in LUAD (i.e. number of samples with at least one deleterious mutation site/number of samples with SNV mutation data) (**Figure 2C**). We then the CNVs in the CRGs and found associations between CNVs and mRNA levels of nine genes (*DLD*, *LIAS*, *PDHB*, *DLAT*, *MTF1*, *CDKN2A*, *FDX1*, *PDHA1*, and *LIPT1*) (**Figure 2D**). Homogeneous and heterogeneous variations, that is, homogeneous/heterogeneous deletion in *CDKN2A* and heterogeneous amplification in *DLD*/*LIPT1*/*GLS* were found (**Figure 2E**). **Figure 2F** shows the percentage of various types of CNVs—heterozygous amplification, heterozygous deletion, homozygous amplification, and homozygous deletion—in each CRG in LUAD. Methylation is an important epigenetic alteration that remodels genes, including those associated with cancer and may lead to uncontrolled growth. Therefore, we investigated the methylation profiles of CRGs in LUAD samples and assessed corresponding mRNA expression. We found that the methylation levels of CRGs were largely negatively correlated with mRNA levels (**Figure 2G**). In addition, six genes in LUAD (*LIPT1*, *DLAT*, *DLD*, *PDHA1*, *GLS*, and *CDKN2A*) had different methylation levels (**Figure 2H**). As *CDKN2A*, a pivotal contributor in the cell cycle, showed the highest ectopic expression, deleterious mutation frequency, and differentiated CNV in LUAD compared with normal controls, we tried to verify its expression in LUAD tissues. We measured *CDKN2A* expression in five pairs of tissues using RT-qPCR and found that *CDKN2A* expression was significantly higher in LUAD compared to normal lung tissue (**Figure 2I**). Immunohistochemistry results also showed that CDKN2A expression was higher in LUAD tissues compared to normal lung tissues (**Figure 2J**). In summary, our analysis revealed significant differences in the genetic profiles and expression of CRGs between LUAD and control samples, suggesting a potential role of CRGs in LUAD tumorigenesis and progression.

### Construction of comprehensive LUAD cohort

**Figure 3:**
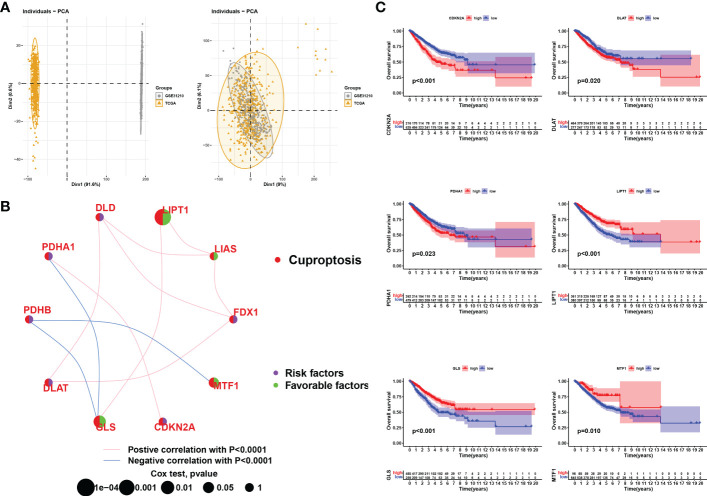
Correlation analysis and survival analysis of CRGs in LUAD. **(A)** Data from LUAD in TCGA were merged with LUAD in GSE31210. Left: PCA plot before the batch effect is removed, right: PCA plot after the batch effect is removed. **(B)** Interaction of CRGs in LUSD. The size of the circles represents the prognostic impact of each CRGs. The green dots in the circles represent protective factors and the purple represents risk factors. The links between genes represent their interactions, the pink line represents positive correlations, the blue line represents negative correlations and the thickness of the line represents the strength of the correlation between them, p<0.0001. **(C)** KM survival curves for each cuproptosis-related gene (only those with p<0.05 are shown).

To investigate the potential biological functions of cuproptosis in LUAD tumorigenesis in a larger sample, we merged the TCGA-LUAD samples and the GSE31210 dataset. After batch effects removal using the R packages “limma” and “sva”, we obtained a comprehensive cohort of 750 LUAD samples and 16,928 genes (**Figure 3A**). In addition, we constructed a network map reflecting the correlation of ten CRGs with clinical outcomes, and manifesting the molecular interactions between these CRGs (**Figure 3B**). We then performed the Kaplan-Meier analysis to investigate the prognostic value of the CRGs in LUAD (**Figure 3C**). Survival curves revealed that PDHA1, DLAT, and CDKN2A were risk factors, and LIPT1, GLS, and MTF1 were protective factors in LUAD. In conclusion, we found that CDKN2A, DLAT, and PDHA1 were not only highly expressed in LUAD samples but also predicted poor clinical outcomes in the long term.

### Identification and evaluation of subtypes

**Figure 4:**
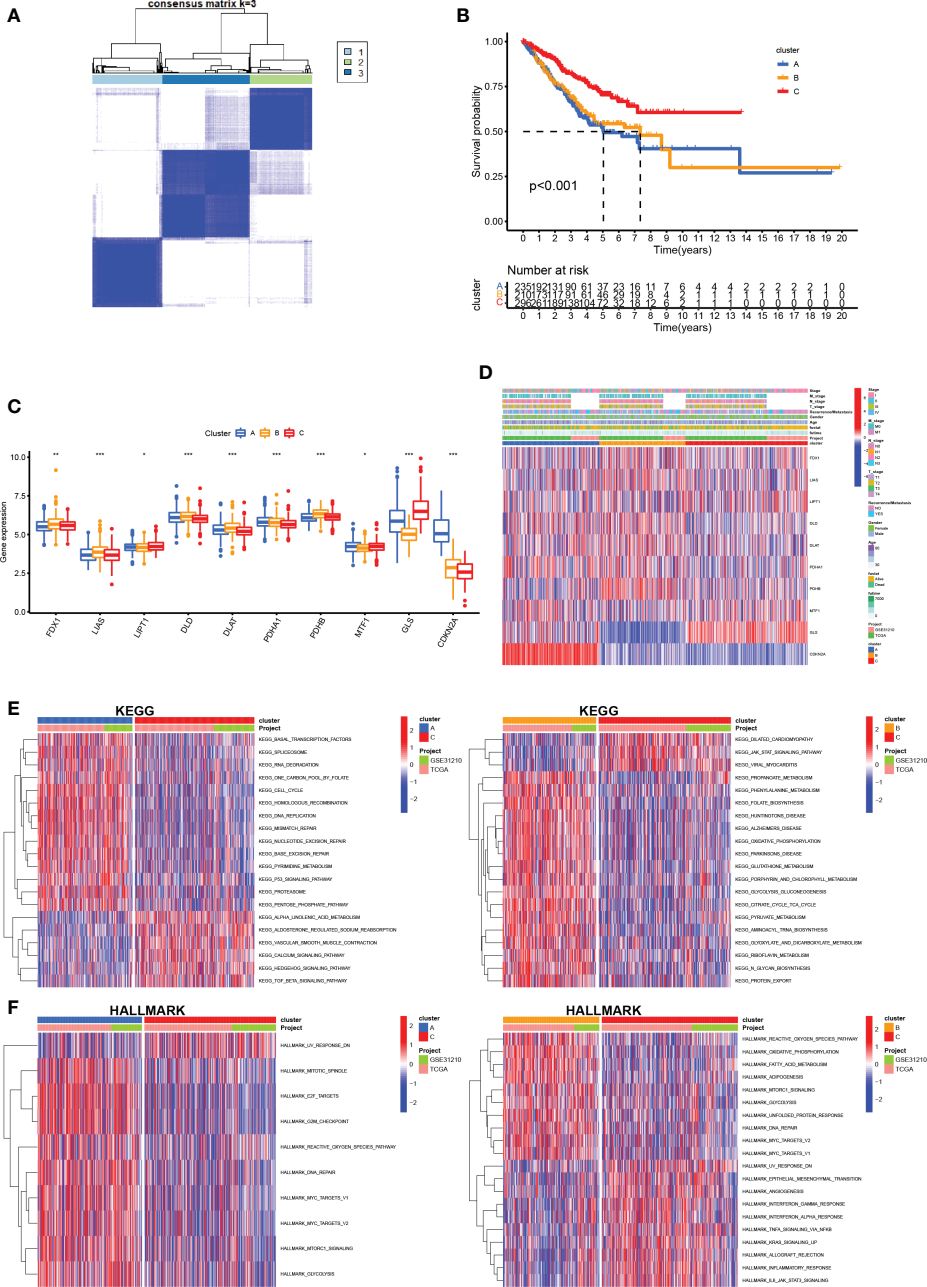
Construction of CRG subtypes **(A)** Consensus matrix heatmap defining three subtypes (k = 3) and their correlation area. **(B)** OS curves for the three cuproptosis-related subtypes based on 750 patients with LUAD from two cohorts (TCGA and GSE31210). **(C)** Differential expression of CRGs between different subtypes. **(D)** Differences in clinicopathologic features and expression levels of CRGs between the three distinct subtypes. **(E, F)** GSVA of KEGG and HALLMARK pathways between distinct subtypes, in which red and blue represent activated and inhibited pathways, respectively. *P <.05; **P <.01; ***P <.001.

We performed unsupervised clustering and classification based on the CRGs. By increasing the clustering variable (k) from 2 to 10, we found the highest intra-group correlations and low inter-group correlations at k = 3, suggesting that LUAD patients may be well segregated into three clusters (**Figure 4A**). Survival analysis revealed that prognosis differed substantially among the three cuproptosis subtypes, and subtype C had considerable survival advantages (p < 0.001, **Figure 4B**). Subtype C had a longer survival time compared to subtype A and subtype B. Moreover, the expression of CRGs in the different clusters also showed significant differences (**Figure 4C**). The clinicopathological features of the three subtypes and the expression of the CRGs were unveiled using a heat map (**Figure 4D**).

To further infer the biological characteristics of the more malignant LUAD cluster, we compared the pathways enriched in each of the LUAD subclusters. We downloaded the KEGG pathway and the HALLMARK pathway from the Msigdb database and subsequently performed GSVA scoring of these pathways and compared the differences in the pathways enriched in each of the three cuproptosis mutant subtypes (**Figures 4E, F**). We found significant differences between the three subtypes, mainly in metabolism-related pathways (propanoate metabolism, phenylalanine metabolism, fatty acid metabolism, and glutathione metabolism) and immune-related pathways (IL6/JAK/STAT3, inflammatory response, interferon alpha response, and interferon-gamma response).

### Anti-tumor effect of cuproptosis in LUAD

**Figure 5:**
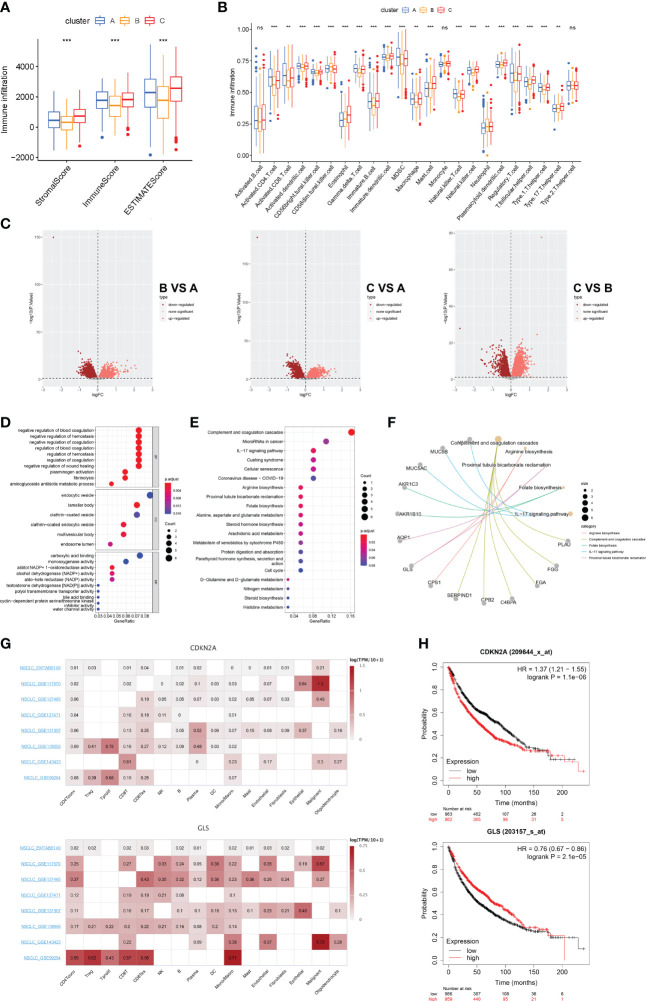
Landscape of biological characteristics of cuproptosis-related subtypes and gene **(A)** Correlations between the three cuproptosis subtypes and TME score. **(B)** The abundance of 23 kinds of infiltrating immune cells was evaluated by ssGSEA in the three cuproptosis subtypes. **(C)** The results of the differences between the three distinct subtypes are shown by the volcano map. **(D)** GO enrichment analyses of DEGs among three distinct subtypes. **(E)** KEGG enrichment analyses of DEGs among three distinct subtypes. **(F)** Correspondence between genes and pathways in the top 5 KEGG results. **(G)** Single-cell expression of CRGs in 8 independent datasets (NSCLC METAB6149, NSCLC GSE117570, NSCLC GSE127465, NSCLC GSE127471, NSCLC GSE131907, NSCLC GSE139555, NSCLC GSE143423, and NSCLC GSE99254). **(H)** Prognostic analysis of CDKN2A and GLS in 1925 LUAD clinical samples. ns, no significant difference, **p < 0.01; and ***p < 0.001.

The aforementioned findings suggest that 1) cuproptosis is associated with the malignancy and tumorigenesis of LUAD and that 2) LUAD subclusters grouped by cuproptosis-related mechanisms are differentiated in anti-tumor immunity. Therefore, we sought to quantify the infiltration landscape of the tumor microenvironment (TME) in patients with LUAD. We assessed TME scores (stromal score, immune score, and estimated score) of the three subtypes using the “ESTIMATE” package. For the TME score, a higher stromal score or immune score represents a higher relative abundance of stromal or immune cells in the TME, while the estimated score indicates the aggregation of stromal or immune scores in the TME. The results showed that in all three cuproptosis subtypes, patients with subtype C had the highest TME scores (**Figure 5A**). The ssGSEA algorithm was used to calculate the fraction of immune cell infiltration per LUAD sample between the three subtypes and to compare the differences in immune cell infiltration between the subtypes. We observed significant differences in the infiltration of most immune cells between the three subtypes (**Figure 5B**). The results showed that compared with clusters A and B, cluster C had more activated dendritic cells, CD56 bright natural killer cells, eosinophils, immature B cells, immature dendritic cells, macrophages, mast cells, monocytes, natural killer cells, plasmacytoid dendritic cells, T follicular helper cells, Type 1 T helper cells, and Type 17 T helper cells. This also explains why cluster C had the best survival advantage. With these results, we found that subtype C had better TME scores, immune infiltration levels, and LUAD prognosis. To further explore the potential feature of each cuproptosis subtype, we performed a differential analysis of the three cuproptosis subtypes. Discrepant analysis results of the three cuproptosis subtypes are summarized in **Supplementary Table 1** and the DEGs are shown using Volcano plots (p<0.05, **Figure 5C**). Notably, *CDKN2A* was significantly more expressed in subcluster A and subcluster B, while *GLS* was significantly more expressed in subcluster C, indicating a potential positive correlation between high *CDKN2A* and low *GLS* expression and worse clinical outcomes of LUAD. We then screened DEGs with a log fold change> 7 and identified the 72 most significantly differentially expressed genes among the three cuproptosis-related LUAD subtypes. GO (**Figure 5D**) and KEGG (**Figure 5E**) enrichment analyses were performed to identify the top five pathways associated with the DEGs based on adjusted p-value in KEGG analysis and their relationship networks with related genes were displayed (**Figure 5F**). The results suggested that the CRGs were mainly associated with processes related to aberrant metabolism and tumor immunity. Of the top 5 enriched pathways, immune-related processes, such as complement and coagulation cascades and IL-17 signaling pathways were further studied.

To validate our findings, we extended our sample size and covered eight single-cell transcriptomic datasets (EMTAB6149, GSE117570, GSE127465, GSE127471, GSEGSE131907, GSE139555, GSE143423, and GSE99254) to assess the correlation between CRGs and TME cellular components after cell annotation. We found that compared with *CDKN2A, GLS* was significantly highly expressed in several immune clusters including CD4+T, CD8+T, and mononuclear phagocytosis system (MPS) derived from the LUAD TME. (**Figure 5G**). We then investigated the prognostic relevance of *CDKN2A* and *GLS* expression in patients with LUAD. We analyzed 1925 LUAD clinical data using the Kaplan-Meier Plotter database and found that patients in the low CDKN2A/high GLS expression group had a better prognosis than those in the high CDKN2A/low GLS expression group (**Figure 5H**). These findings suggest that cuproptosis could serve as an indicator to predict tumor immunity and prognosis.

### Constructing the cuproptosis score

**Figure 6:**
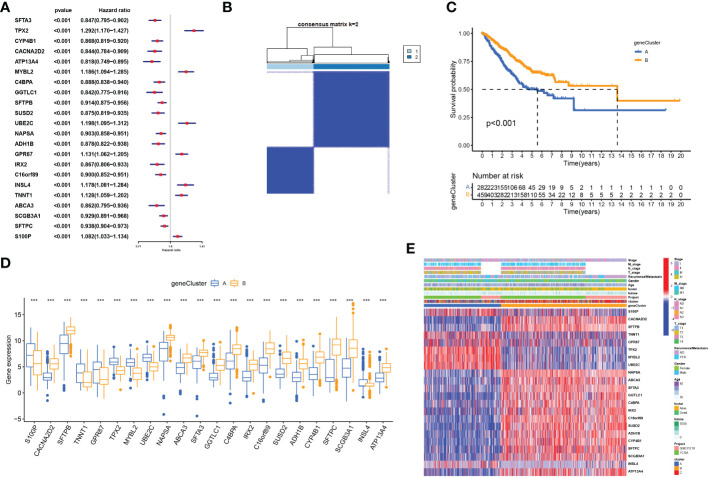
Identification of gene subtypes based on cuproptosis subtype-related DEGs **(A)** Univariate regression analysis of the 22 cuproptosis subtype-related DEGs, pvalue < 0.001. **(B)** Consensus matrix heatmap defining two clusters (k = 2) and their correlation area. **(C)** OS curves for the two gene subtypes. **(D)** Differential expression of 22 cuproptosis subtype-related DEGs between two gene subtypes. **(E)** Differences in clinicopathologic features and expression levels of 22 cuproptosis subtype-related DEGs between two gene subtypes, ***P <.001.

To identify CRGs with prognostic significance in LUAD, we performed a univariate Cox regression analysis on 72 DEGs identified by cuproptosis-related LUAD clusters (**Supplementary Table 2**). Of these, 22 DEGs had prognostic value (p<0.001, **Figure 6A**) and were used for further clustering. At k = 2, intra-group correlations were the lowest, indicating that LUAD patients could be well divided into two gene clusters (**Figure 6B**). Kaplan-Meier curves showed that patients from geneClusterA had worse overall survival (log-rank, p<0.001; **Figure 6C**). As shown in **Figure 6D**, we found significant differences in the expression of the 22 DEGs in these two gene clusters. **Figure 6E** shows the gene expression profiles as well as clinicopathological features of the 22 DEGs in the two gene subtypes.

### Cuproptosis score for predicting clinical outcomes and the TIME

**Figure 7:**
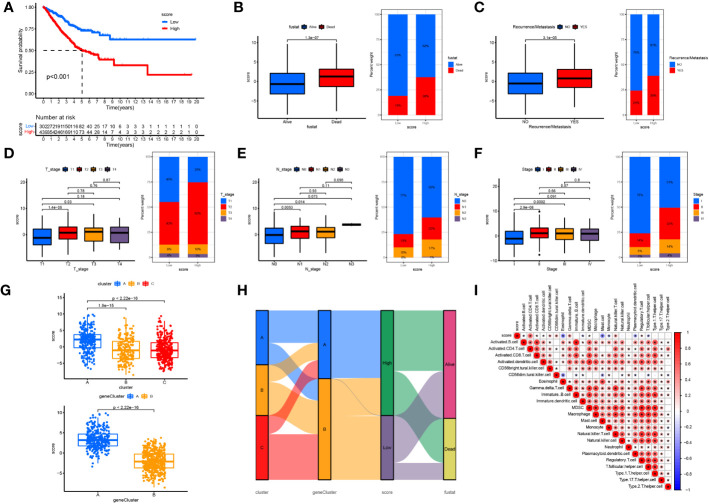
Constructing the cuproptosis score in predicting clinical outcomes and TIME **(A)** OS curves for the high- and low-cuproptosis score patients. **(B)** Relationships between cuproptosis score and survival status. **(C)** Relationships between cuproptosis score and recurrence/metastasis. **(D)** Relationships between cuproptosis score and T-stage. **(E)** Relationships between cuproptosis score and N-tage. **(F)** Relationships between cuproptosis score and recurrence/TMN-tage. **(G)** The relationship between the cuproptosis score and cuproptosis clusters **(A-C)** and cuproptosis gene clusters **(A, B)**. **(H)** The Sankey diagram was drawn to illustrates the distribution of patients in three cuproptosis subtypes, two gene subtypes, two cuproptosis score groups and survival status. **(I)** The correlation between the score and immune cell infiltration was calculated, with red representing a positive correlation and blue a negative correlation, the darker the colour the stronger the correlation. *P <.05.

To further evaluate the association between cuproptosis and LUAD prognosis, we constructed a prognostic signature for cuproptosis. After dimensional reduction, we defined the Cuproptosis score that predicts the long-term prognosis as the sum of transcriptomic profiles derived from two geneClusters. LUAD patients with higher Cuproptosis scores were divided into the high-risk group and those with low Cuproptosis scores, in the low-risk group. Kaplan-Meier survival curves showed a significant difference between the two groups and verified the correlation between cuproptosis-based transcriptomic profiles and LUAD prognosis (log-rank test, p<0.001; **Figure 7A**). We then explored the correlation between the Cuproptosis score and the clinicopathological features of LUAD. We observed that patients in the high-risk group were associated with higher mortality, TNM staging, and recurrent/metastatic tumors (**Figures 7B–F**). The relationship between the Cuproptosis score and cuproptosis clusters A-C and cuproptosis geneClusters A-B was further explored (**Figure 7G**). Cluster A and gene cluster A were significantly correlated with a high Cuproptosis score and a worse prognosis. It was proved that prognostic results obtained by different clustering modes were consistent. Subsequently, a Sankey diagram was drawn to illustrate the relationship between patients in the three cuproptosis subtypes, two gene subtypes, two cuproptosis score groups, and survival status (**Figure 7H**). Finally, tumor tissues from the high-risk group were enriched with immunosuppressive cells such as Treg and MDSC and lacked effector cells such as activated B cells, activated dendritic cells, eosinophils, immature dendritic cells, mast cells, monocytes, plasmacytoid dendritic cells, and T follicular helper cells (**Figure 7I**). These findings indicate that cuproptosis-based transcriptomic characteristics could contribute to identifying LUAD cases with poor immune infiltration and a high likelihood of poor clinical outcomes.

### Cuproptosis score for predicting sensitivity to immunotherapy and targeted drugs

**Figure 8:**
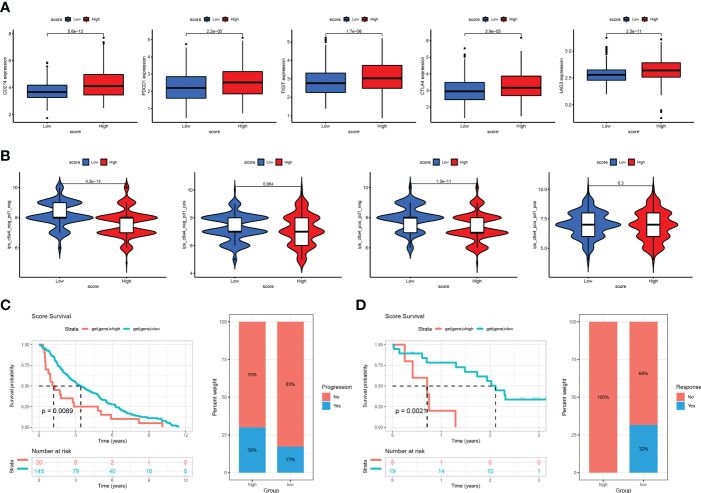
The association between cuproptosis score and immunotherapy response **(A)** Correlations between risk score and PD1, PD-L1, CTLA4, LAG3, and TIGIT. **(B)** Immunophenoscore (IPS) function is based on The Cancer Immunome Atlas database to predict the responsiveness to CTLA-4 and PD-1. **(C)** The Kaplan-Meier OS and percentage of responsive analysis of cuproptosis score in GSE91061 anti-CTLA4 pre-therapy cohort (Nivolumab immunotherapy). **(D)** The Kaplan-Meier OS and percentage of progressive analysis of cuproptosis score in GSE13507 cohort (intravesical BCG immunotherapy).

After constructing the Cuproptosis score and verifying its utility in prognosis prediction and reflecting immune infiltration, we explored whether this score could be applied to facilitate immunotherapy in clinical practice. First, we found that the expression of most immunosuppressive genes (such as *PD1*, *PD-L1*, *CTLA4*, *LAG3*, *CTLA4*, and *TIGIT*) was significantly higher in the high- than in the low-risk group (**Figure 8A**). We then used two methods to validate the ability of the Cuproptosis score to predict the benefits of immunotherapy. Recent studies have reported that IPS based on immunogenicity could effectively predict the response to immunotherapy in patients with malignancies (10). Therefore, we downloaded the IPS of the TCGA-LUAD cohort from TCGA database to explore differences in the efficacy of immunotherapy between the high- and low-cuproptosis groups. Then, we compared the correlation between the IPS and the Cuproptosis score. In the immunotherapy scoring, we found that both anti-PD-1 and anti-CTLA-4 resulted in better immunotherapy benefits for patients and that compared to patients in the high cuproptosis group, those in the low cuproptosis group benefited more from immunotherapy (**Figure 8B**). To further analyze the application of the cuproptosis score in the assessment of immunotherapy, we used the GSE91061 and GSE13507 cohorts, in which patients received nivolumab and intravesical BCG immunotherapy, to be divided into the high Cuproptosis score and low Cuproptosis score groups. Through Kaplan-Meier analysis, we found that patients with a low Cuproptosis score had a better prognosis and higher response percentage than high Cuproptosis score (**Figures 8C, D**). These results also reaffirmed that patients with low Cuproptosis scores benefit more from immunotherapy than those with high Cuproptosis scores and that the combination of the Cuproptosis score with ICI expression may help better predict sensitivity to immunotherapy. In conclusion, the Cuproptosis score has great potential in predicting prognosis and immunotherapeutic benefits.

**Figure 9:**
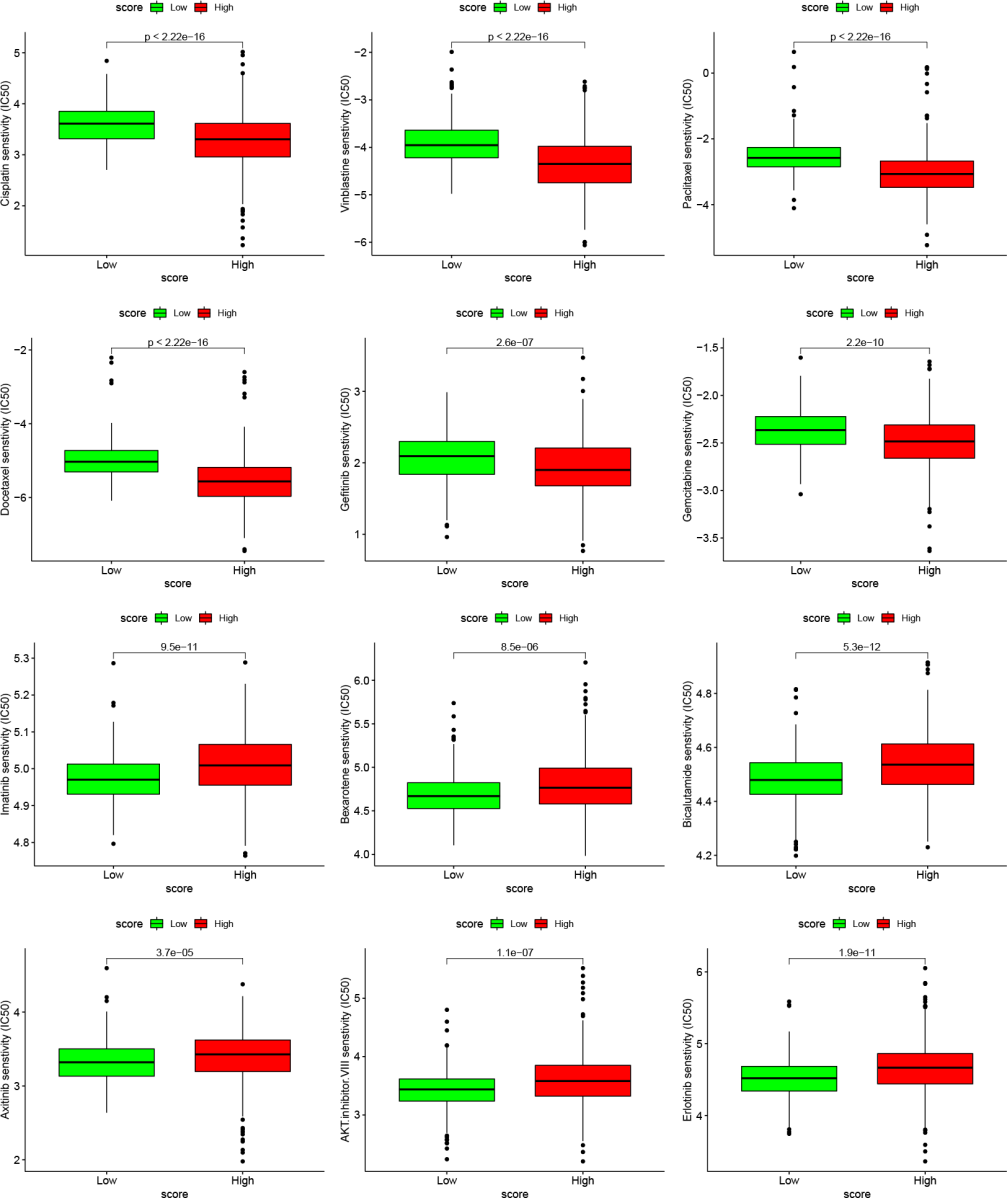
Relationships between cuproptosis score and chemotherapeutic sensitivity Box plots based on the estimated IC50 of the 12 compounds.

Finally, we selected the targeted drugs currently used to treat LUAD to assess the sensitivity of these agents in patients grouped by Cuproptosis score. The “pRRophetic” package was used to predict the IC50 values for each sample for multiple targeted drugs, comparing the differences between the high- and low-score groups. In patients with a high cuproptosis score, we predicted low IC50 values and high sensitivity to targeted drugs, such as cisplatin, vinblastine, paclitaxel, docetaxel, gefitinib, and gemcitabine. In patients with a low cuproptosis score, we predicted low IC50 values and high sensitivity to targeted drugs such as imatinib, bexarotene, bicalutamide, axitinib, AKT inhibitor VIII, and erlotinib (**Figure 9**). These results show that the Cuproptosis score can predict not only prognosis and immunotherapeutic benefits but also guide targeted drugs and comprehensive patient treatment. It is a novel tumor biomarker with outstanding potential.

## Discussion

Copper is an indispensable microelement involved in various biological processes, and dysregulation of copper homeostasis is closely associated with the development of several tumors. Recent studies have linked elevated copper levels in both serum and tumor samples of various patients, as well as, imbalanced copper metabolism and dysregulated oxidative stress with tumor progression (11–14). Consequently, copper ion carriers (disulfiram, dithiocarbamate, eschlorophen, etc.) and copper chelators (tretinoin, tetrathiomolybdate, etc.) have been used effectively in anticancer therapy (15–18). Cuproptosis, a copper-mediated cell death pathway, is expected to provide new strategies for predicting the prognosis and treatment of patients with LUAD.

In this study, we comprehensively evaluated the transcriptional expression levels, genetic alterations, prognostic value, and immune landscape of CRGs in LUAD. First, we found the bilateral effects of cuproptosis biomarkers in LUAD samples. We found increased expression of CDKN2A, DLAT, LIAS, DLD, and PDHA1 and decreased expression of MTF1 and FDX1 in LUAD tissues compared to normal lung tissues. *CDKN2A* was identified as a gene that is not only overexpressed gene but harbors deleterious mutations linked to LUAD. According to previous studies, the loss or mutation in *CDKN2A* leads to uncontrolled cancer cell proliferation, while TP53 mutations are associated with CDKN2A mutations and high tumor mutational burden (19, 20). Thus, CDKN2A plays a critical role in tumorigenesis and progression. Furthermore, we identified three cuproptosis subtypes based on seven differentially expressed CRGs, showing different prognoses. To explore the mechanisms underlying these findings, we used GSVA enrichment analysis and demonstrated that cuproptosis-based bioinformatic analysis functioned well in differentiating more proliferative LUAD cancers and patients with better prognoses. Considering the impact of cuproptosis on LUAD heterogeneity and corresponding clinical outcomes, we further constructed two gene subtypes based on 22 cuproptosis subtype-related DEGs and used the Boruta algorithm combined with PCA to construct a Cuproptosis score. The subsets with the poorest prognosis all had relatively high Cuproptosis scores. Patients with a low Cuproptosis score had a higher survival rate than those with a high Cuproptosis score, demonstrating that the established Cuproptosis score is valid for the primary prediction of LUAD prognosis. In addition, patients with a high Cuproptosis score had worse clinical outcomes in that they had higher mortality, TNM staging, and recurrence and metastasis.

To investigate the potential mechanism by which cuproptosis status mediates LUAD prognosis, we assessed the TIME of LUAD tissues. scored compared with LUAD patients from other clusters, those from cluster A had a higher cuproptosis score, indicating immune cell deficiency in the TME. Moreover, the Cuproptosis score served as an effective predictor to identify immunosuppression. Immune checkpoint inhibitors (ICIs) represented by PD-1/PDL1, CTLA4, TIGIT, and LAG3 were significantly expressed in the group with a high cuproptosis score, which indirectly suggests that the cuproptosis score may play an important role in predicting immunotherapy success (21, 22). Disturbed copper homeostasis affects cellular biology in several ways such as posttranscriptional regulation, cell membrane system disorder, oxidative stress, and mitochondrial damage (23). It also causes genomic instability and pro-tumoral mutation, as well as, contributes to uncontrollable proliferation and ectopic phenotypes of cancer cells, increasing sensitivity to chemotherapy and immune cell infiltration in the tumor sites. Therefore, we used two methods to validate the ability of the Cuproptosis score to predict the benefits of immunotherapy and confirmed that patients with low Cuproptosis scores benefit better from immunotherapy than those with high Cuproptosis scores due to better immune cell infiltration. We suggest that the integrated use of the Cuproptosis score with ICI expression may help effectively predict the sensitivity to immunotherapy. The Cuproptosis score we established has great potential to predict prognosis and immunotherapy efficacy.

As this study was constructed using retrospective datasets of public transcriptomics, further *in vivo* experiments are needed to better consolidate bioinformatic analyses, and large prospective studies and additional *in vivo* and *in vitro* experimental studies are needed to confirm our findings.

## Conclusion

Our comprehensive analysis revealed that cuproptosis is an underlying regulator of LUAD progression and prognosis. The identification of cuproptosis-based LUAD subtypes will help gain insights into the heterogeneity of LUAD. Moreover, we constructed a cuproptosis scoring system that serves as a promising indicator to determine the prognosis, clinical outcomes, TME characteristics, and immunotherapy success of patients with LUAD. The findings of our study offer insights into the development of novel strategies for the diagnosis, prognosis evaluation, and treatment of patients with advanced LUAD.

## Data availability statement

The original contributions presented in the study are included in the article/**Supplementary Material**. Further inquiries can be directed to the corresponding author.

## Ethics statement

TCGA belong to public databases. The patients involved in the database have obtained ethical approval. Users can download relevant data for free for research and publish relevant articles. Our study is based on open source data, so there are no ethical issues and other conflicts of interest. The Ethics Committee of local legislation and institutional has granted exemptions from approval for research related to the use of such public databases.

## Author contributions

GW, QH, and HR acquired the data. GW and QH wrote the whole manuscript. MH and HM performed statistical analysis and technical support. LZ and KX conceived the idea and designed the study. HC and JQ made significant revisions to the manuscript and finally approved for publication. All authors contributed to the article and approved the submitted version.
